# A Fast Multiple Change-Point Detection Method via Generalized Nearly Isotonic Optimization

**DOI:** 10.3390/e28090961

**Published:** 2026-08-27

**Authors:** Luoxin Wang, Mengmeng Wang, Baisuo Jin, Yuehua Wu

**Affiliations:** 1 Department of Statistics and Finance, University of Science and Technology of China, Hefei 230026, China; wwwang0903@mail.ustc.edu.cn (L.W.); jbs@ustc.edu.cn (B.J.); 2 College of Mathematics and System Science, Xinjiang University, Urumqi 830017, China; 107552403374@stu.xju.edu.cn; 3 School of Mathematical Sciences, Xinjiang Normal University, Urumqi 830017, China; 4 Department of Mathematics and Statistics, York University, Toronto, ON M3J 1P3, Canada

**Keywords:** multiple change-point detection, *O*(*n*) complexity, dynamic programming, generalized nearly isotonic optimization

## Abstract

In this paper, we study the generalized nearly isotonic optimization (GNIO) model and its dynamic programming solution (GNIO-DP). We introduce randomness into the GNIO-DP algorithm, enabling its first application to change-point detection and resulting in an O(n) complexity multiple change-point detection method. At the same time, we provide the theoretical properties of the change-point detection and prove the reliability and effectiveness of the GNIO-DP algorithm for this task. The simulation results show that our method has strong change-point detection ability. Compared with traditional methods, our method is faster in most scenarios.

## 1. Introduction

With the rapid growth in the size of modern datasets, detecting changes in the distributional characteristics of data sequences has become increasingly important across a wide range of applications. For example, in physics [1], change-point detection methods can be applied to analyze seismicity. In finance [2], change-point analysis can be used to study the time-varying covariance structure of multivariate financial data. In bioinformatics [3], it can help to identify genes that are associated with specific diseases. Additionally, change-point analysis is useful for detecting credit card fraud [4] and other anomalies [5,6], as well as classifying data in data mining [7]. Furthermore, it has applications in signal processing to detect significant changes in image streams [8]. In many practical situations, such data sequences contain multiple change-points, creating strong demand for efficient and reliable change-point detection methods. It is the primary focus of this paper, where we aim to develop a fast change-point detection method under the conventional assumption of multiple change-points.

Among the existing methods, binary segmentation, originally proposed by [9], remains one of the most widely used approaches for change-point detection. As an approximate method, it has a relatively low computational complexity of O(nlogn), where *n* denotes the number of observations. Wild binary segmentation (WBS), proposed by [10], and narrowest-over-threshold (NOT), proposed by [11], are both improvements over the classical binary segmentation. In contrast, exact change-point detection algorithms usually require higher computational complexity, which is typically on the order of $O(n^{2})$ [12,13]. This makes them prohibitively slow for large datasets. Notably, [14] proposed an algorithm called pruned exact linear time (PELT), which reduces the complexity to O(n) but under the assumption of change-points being separated by time intervals drawn independently from a probability distribution, a setup under which considerations of statistical consistency are impossible due to these spacings being too short. However, in the worst case, the computational complexity of PELT remains $O(n^{2})$. In this paper, we extend the dynamic programming algorithm proposed in [15] to detect change-points in mean-shift models, achieving a computational complexity of O(n). Compared with PELT, our method is stable under the conventional assumption of multiple change-points, and its computational complexity does not revert to $O(n^{2})$. The theoretical guarantees for these properties are established in Theorems 1–3.

The generalized nearly isotonic optimization (GNIO) model was proposed in [15], which recovers, as special cases, many classic problems in shape-constrained statistical regression, such as isotonic regression, nearly isotonic regression and unimodal regression problems. Its exact dynamic programming solver (GNIO-DP) has a computational complexity of O(n) for the $l_{2}$-GNIO problem. This paper aims to adapt the GNIO-DP algorithm for change-point detection through appropriate parameter selection, thereby obtaining a change-point detection method with O(n) computational complexity. Notably, the existing work on GNIO-DP is primarily optimization-oriented and treats the input vector as fixed. However, in the context of statistical change-point detection, the observed data are random. The solution of GNIO-DP should therefore be viewed as a random function of the observed sample.

Our work makes two main contributions. First, we introduce randomness into the GNIO-DP algorithm, enabling its first application to change-point detection and resulting in an O(n) complexity change-point detection method. Second, we provide the theoretical properties of the change-point detection and prove the reliability and effectiveness of the GNIO-DP algorithm for this task. The simulation results show that our method has strong change-point detection ability. Compared with traditional methods, our method is faster in most scenarios.

This paper is organized as follows. Section 2 starts by introducing the basic notation for change-points and summarizes the relevant literature. Section 3 presents the computational complexity and theoretical properties of the GNIO-DP algorithm for change-point detection. Section 4 provides the simulation results. Section 5 applies our method to analyze the well-log data. Section 6 concludes the paper.

## 2. Background

### 2.1. Model

This work primarily focuses on the computational complexity of the proposed method. For simplicity, we describe and analyze the method for the well-studied detection of shifts in means [10,11,14]. However, the applicability of this method goes far beyond this simple setup. Formally, consider an ordered sequence of data $y={\left(y_{1},\ldots ,y_{n}\right)}^{⊤}$ for i=1,…,n,

(1) $$y_{i}=\theta_{i}^{*}+\epsilon_{i},$$

where $\theta_{i}^{*}$ is a fixed one-dimensional piecewise constant signal with an unknown number *m* of change-points at unknown locations $0=\tau_{0}<\tau_{1}<\cdots <\tau_{m}<\tau_{m+1}=n$; that is,

$$\theta_{1}^{*}=\cdots =\theta_{\tau_{1}}^{*}\neq \theta_{\tau_{1}+1}^{*}=\cdots =\theta_{\tau_{2}}^{*}\neq \theta_{\tau_{2}+1}^{*}=\cdots \cdots \neq \theta_{\tau_{m}+1}^{*}=\cdots =\theta_{n}^{*}.$$

The errors $\epsilon_{i}$, i=1,…,n, are assumed to be independent and identically distributed sub-Gaussian random variables with sub-Gaussian norm ${∥\epsilon_{i}∥}_{\psi_{2}}=\sigma$, where

$${∥X∥}_{\psi_{2}}=\inf\left\{t>0:E\exp\left(X^{2}/t^{2}\right)\leq 2\right\}$$

for any random variable *X*.

**Remark** **1.** *The assumption of independent and identically distributed errors is imposed primarily to facilitate the theoretical analysis. In contrast, the GNIO-DP algorithm itself does not explicitly require the errors to be independent and therefore remains applicable under dependent error structures. Nevertheless, the consistency results established in Theorems 2 and 3 rely on the concentration properties induced by the i.i.d. sub-Gaussian error assumption and would require additional conditions to accommodate dependence, such as suitable weak-dependence or mixing conditions. Establishing theoretical guarantees for GNIO-DP under dependent error structures is an interesting direction for future research.*

### 2.2. Generalized Nearly Isotonic Optimization

This subsection introduces the fundamental structure of the generalized nearly isotonic optimization (GNIO) model and its dynamic programming solution (GNIO-DP), laying the groundwork for the subsequent statistical theory. Building upon the GNIO-DP algorithm proposed by [15], this work enables its first application to change-point detection and results in an O(n) complexity change-point detection method.

#### 2.2.1. GNIO Problem and Notation

We address the following GNIO problem:

(2) $$\min_{\theta \in R^{n}}\left\{\sum_{i=1}^{n}f_{i}\left(\theta_{i}\right)+\sum_{i=1}^{n-1}\lambda_{i}{\left(\theta_{i}-\theta_{i+1}\right)}_{+}+\sum_{i=1}^{n-1}\mu_{i}{\left(\theta_{i+1}-\theta_{i}\right)}_{+}\right\},$$

where ${\left(a\right)}_{+}:=\max\left\{0,a\right\}$ for any a∈R, and the regularization parameters satisfy

$$0\leq \lambda_{i},\;\mu_{i}\leq +\infty ,\;i=1,\ldots ,n-1.$$

If $\lambda_{i}=+\infty$ (respectively, $\mu_{i}=+\infty$) for some *i*, the corresponding term $\lambda_{i}{\left(\theta_{i}-\theta_{i+1}\right)}_{+}$ (respectively, $\mu_{i}{\left(\theta_{i+1}-\theta_{i}\right)}_{+}$) is interpreted as the hard constraint $\theta_{i}\leq \theta_{i+1}$ (respectively, $\theta_{i}\geq \theta_{i+1}$).

Throughout this paper, we focus on the convex loss

(3) $$f_{i}\left(\theta_{i}\right)=f\left(\theta_{i},y_{i}\right),\;i=1,\ldots ,n,$$

where $y_{i}\in R$ are given observations. Under (2) and (3), the GNIO model can be viewed as a mapping from the observation vector $y={\left(y_{1},\ldots ,y_{n}\right)}^{⊤}$ to the minimizer $\hat{\theta }\left(y\right)={\left({\hat{\theta }}_{1}\left(y\right),\ldots ,{\hat{\theta }}_{n}\left(y\right)\right)}^{⊤}$ of a penalized least-squares criterion. When y follows a mean-shift model, an appropriate choice of parameters $\lambda_{i},\mu_{i}$ allows the change-points to be identified from the solution $\hat{\theta }$.

#### 2.2.2. GNIO-DP Algorithm

To reveal the recursive structure of problem (2), we introduce a family of univariate functions ${\left\{h_{i}\right\}}_{i=1}^{n}$. Define

(4) $$h_{1}\left(\theta_{1}\right)\equiv 0,\;\theta_{1}\in R,$$

and, for i=2,…,n, define $h_{i}$ by

(5) $$h_{i}\left(\theta_{i}\right):=\min_{\theta_{i-1}\in R}\left\{f_{i-1}\left(\theta_{i-1}\right)+h_{i-1}\left(\theta_{i-1}\right)+\lambda_{i-1}{\left(\theta_{i-1}-\theta_{i}\right)}_{+}+\mu_{i-1}{\left(\theta_{i}-\theta_{i-1}\right)}_{+}\right\},\;\theta_{i}\in R.$$

With $g_{i}\left(\theta_{i}\right)=h_{i}\left(\theta_{i}\right)+f_{i}\left(\theta_{i}\right)=h_{i}\left(\theta_{i}\right)+\frac{1}{2}{\left(\theta_{i}-y_{i}\right)}^{2}$, the last coordinate is obtained by minimizing $g_{n}$, and the remaining coordinates are then recovered backward.

Notably, (5) can be derived in the specific form

(6) $$h_{i}\left(\theta_{i}\right)=\left\{\begin{array}{cc} g_{i-1}\left(b_{i}^{-}\right)+\lambda_{i-1}\left(b_{i}^{-}-\theta_{i}\right), & \theta_{i}<b_{i}^{-},\\ g_{i-1}\left(\theta_{i}\right), & b_{i}^{-}\leq \theta_{i}\leq b_{i}^{+},\\ g_{i-1}\left(b_{i}^{+}\right)+\mu_{i-1}\left(\theta_{i}-b_{i}^{+}\right), & \theta_{i}>b_{i}^{+}, \end{array}\right.\;i=2,\ldots ,n,$$

where $b_{i}^{-}=\arg\min_{z\in R}\left(g_{i-1}\left(z\right)+\lambda_{i-1}z\right)$ and $b_{i}^{+}=\arg\min_{z\in R}\left(g_{i-1}\left(z\right)-\mu_{i-1}z\right)$. Correspondingly, the backward recursion can be expressed as

(7) $${\hat{\theta }}_{i}=\min\left(b_{i+1}^{+},\;\max\left(b_{i+1}^{-},\;{\hat{\theta }}_{i+1}\right)\right),\;i=n-1,\ldots ,1.$$

Algorithm 1 outlines the concrete steps of the GNIO-DP algorithm. A detailed discussion of the GNIO-DP algorithm is provided in Appendix A.
**Algorithm 1** GNIO-DP.  1:Initialize $h_{1}\left(\theta_{1}\right)=0$  2:**for** i=2:n **do**  3:    $g_{i-1}=h_{i-1}+f_{i-1}$  4:    $\left(h_{i},b_{i}^{-},b_{i}^{+}\right)=\mathrm{update}\left(g_{i-1},\lambda_{i-1},\mu_{i-1}\right)$ by (6)  5:**end for**  6:$g_{n}=h_{n}+f_{n}$  7:${\hat{\theta }}_{n}=\arg\min_{\theta_{n}\in R}g_{n}\left(\theta_{n}\right)$  8:${\hat{\theta }}_{1},\ldots ,{\hat{\theta }}_{n-1}=\mathrm{recover}\left({\hat{\theta }}_{n},{\left\{(b_{i}^{-},b_{i}^{+})\right\}}_{i=2}^{n}\right)$ by (7)**Ensure:** ${\hat{\theta }}_{1},\ldots ,{\hat{\theta }}_{n}$

Based on (6) and $g_{i}\left(\theta_{i}\right)=h_{i}\left(\theta_{i}\right)+\frac{1}{2}{\left(\theta_{i}-y_{i}\right)}^{2}$, we can obtain ${\left\{b_{i}^{-},b_{i}^{+}\right\}}_{i=2}^{n}$ and the explicit expression for $g_{n}\left(\theta_{n}\right)$. The final fitted value is then obtained as ${\hat{\theta }}_{n}=\arg\min_{\theta_{n}}g_{n}\left(\theta_{n}\right)$. Furthermore, using (7), we can sequentially recover ${\hat{\theta }}_{n-1},\ldots ,{\hat{\theta }}_{1}$. To clarify the underlying dynamic programming procedure, we now describe the roles of the state and decision variables. At step *i*, $\theta_{i}$ is viewed as the state variable, representing the current fitted value, while $\theta_{i-1}$ is the decision variable, representing the fitted value at the previous observation. For a given $\theta_{i}$, $h_{i}\left(\theta_{i}\right)$ represents the minimum objective value obtained by optimizing over the previous fitted value $\theta_{i-1}$. Hence, to update $h_{i}\left(\theta_{i}\right)$, we only need to determine the optimal value of $\theta_{i-1}$ using the information from the previous step rather than reconsidering all the previous fitted values. In summary, the GNIO-DP algorithm can be understood as a two-step procedure: the forward pass efficiently propagates the necessary information from left to right, while the backward pass uses this information to recover the optimal fitted sequence. A simple numerical example is provided in Appendix A to further illustrate these two steps.

## 3. A Fast Multiple Change-Point Detection Method

We now aim to identify the change-points in the sequence $y_{1},\ldots ,y_{n}$ by solving the following $l_{2}$-GNIO problem,

(8) $$\left({\hat{\theta }}_{1},\ldots ,{\hat{\theta }}_{n}\right)=\arg\min_{\theta \in R^{n}}\sum_{i=1}^{n}\frac{1}{2}{\left(\theta_{i}-y_{i}\right)}^{2}+\sum_{i=1}^{n-1}\lambda_{i}{\left(\theta_{i}-\theta_{i+1}\right)}_{+}+\sum_{i=1}^{n-1}\mu_{i}{\left(\theta_{i+1}-\theta_{i}\right)}_{+},$$

where $0\leq \lambda_{i},\mu_{i}\leq +\infty$, i=1,…,n−1. Intuitively, if the sequence is globally stationary (i.e., its mean remains constant), then, under sufficiently large penalties, the GNIO-DP solution is expected to shrink all the adjacent differences to zero and degenerate into an identity constant sequence ${\hat{\theta }}_{1}=\ldots ={\hat{\theta }}_{n}$. Conversely, if the sequence exhibits significant structural changes, the penalty cost of a constant solution becomes excessively high, and more complex piecewise structures will be preferred. Therefore, by appropriately selecting the parameters $\lambda_{i},\mu_{i}$, change-points can be identified based on variations in ${\hat{\theta }}_{1},\ldots ,{\hat{\theta }}_{n}$.

### 3.1. Linear-Time Computational Complexity of GNIO-DP

Based on Section 2.2, we discuss the theoretical computational complexity of GNIO-DP for the $l_{2}$-GNIO problem. As shown in Algorithm 1, the computational complexity of GNIO-DP is primarily determined by (6) and (7). Equation (6) implies that all $g_{i}$ and $h_{i}$ are piecewise quadratic functions. During the recursion, new breakpoints can only arise at $b_{i}^{-}$ and $b_{i}^{+}$, whose specific computation procedures are provided in Appendix A. Once $b_{i}^{-},b_{i}^{+}$, i=2,…,n and ${\hat{\theta }}_{n}$ are known, each evaluation of (7) incurs only O(1) computational cost, repeated n−1 times.

**Theorem** **1.** *The computational complexity of GNIO-DP for solving the $l_{2}$-GNIO problem *(8)* is O(n).*

The detailed derivation of O(n) complexity is provided in Appendix A. PELT also achieves an expected computational complexity of O(n). However, this result is established under the assumption of change-points being separated by time intervals drawn independently from a probability distribution. Under such a framework, the segment lengths do not increase with the sample size *n*, making statistical consistency analysis infeasible. The precise assumptions are given in Theorems 3.1 and 3.2 of [14]. Interestingly, in our simulations, GNIO-DP exhibits faster runtime than the PELT method despite both attaining the same optimal O(n) computational complexity.

### 3.2. Consistency of GNIO-DP for Change-Point Detection

In this subsection, we establish two theorems, which ensure the theoretical validity of applying the GNIO-DP algorithm to change-point detection. First, we make the following reasonable assumption.

**Assumption** **1.** Assume that $\tau_{i}-\tau_{i-1}\geq \Delta$, i=1,…,m+1, with Δ→∞ as n→∞. The magnitudes of the jumps satisfy $\min_{i=1,\ldots ,m}\left|\theta_{\tau_{i}+1}^{*}-\theta_{\tau_{i}}^{*}\right|\geq \delta$. In addition, δ and Δ* are linked by the requirement $\delta^{2}\Delta \geq c\log n$ for a large enough c.*

Assumption 1, adapted from [10], relaxes the traditional condition that the change-point distance Δ is of the same order as *n*.

**Theorem** **2.** *Assume that the data are generated from model *(1)* with m=1 and that the penalty parameters $\lambda =\lambda_{i}=\mu_{i}$, i=1,…,n−1 satisfy*

$$c_{1}\sqrt{\Delta^{2-\alpha }\log\Delta }\leq \lambda \leq c_{2}\Delta^{\beta }\delta ,$$

*where $c_{1},c_{2}>0$ are constants, 0<α,β<1, $\delta =o(\Delta^{1-\beta })$, and $\Delta^{2\beta -\max(\alpha ,\beta )}\delta^{2}\to \infty$ when n→∞. The estimates ${\hat{\theta }}_{1},\ldots ,{\hat{\theta }}_{n}$ are obtained by solving the $l_{2}$-GNIO problem *(8)*. Then, under Assumption 1, there exists a constant $c_{3}>0$ such that*

(9) $$\begin{array}{cc} \; & \lim_{n\to \infty }P\left({\hat{\theta }}_{1}=\cdots ={\hat{\theta }}_{\left\lfloor \tau_{1}-c_{3}\Delta^{\alpha }\right\rfloor }\in \left[{\bar{y}}_{1:q_{1}}-\frac{\lambda }{q_{1}},{\bar{y}}_{1:q_{2}}+\frac{\lambda }{q_{2}}\right]\right)=1,\\ \; & \lim_{n\to \infty }P\left({\hat{\theta }}_{\left\lceil \tau_{1}+c_{3}\Delta^{\beta }\right\rceil }=\cdots ={\hat{\theta }}_{n}\in \left[{\bar{y}}_{q_{3}:n}-\frac{\lambda }{n-q_{3}+1},{\bar{y}}_{q_{3}:n}+\frac{\lambda }{n-q_{3}+1}\right]\right)=1,\\ \; & \lim_{n\to \infty }P\left({\hat{\theta }}_{\left\lceil \tau_{1}-c_{3}\Delta^{\alpha }\right\rceil }=\cdots ={\hat{\theta }}_{\left\lfloor \tau_{1}+c_{3}\Delta^{\beta }\right\rfloor }\right)=0, \end{array}$$

*where $q_{1},q_{2}\in \left[\left\lfloor \tau_{1}-c_{3}\Delta^{\alpha }\right\rfloor ,\tau_{1}\right]$, $q_{3}\in \left[\tau_{1}+1,\left\lceil \tau_{1}+c_{3}\Delta^{\beta }\right\rceil \right]$, and ${\bar{y}}_{a:b}=\sum_{i=a}^{b}y_{i}/\left(b-a+1\right)$ for 1≤a≤b≤n. Furthermore, both interval lengths in (9) are of order O(λ/Δ)=o(1).*

**Theorem** **3.** *Assume that the data are generated from model *(1)* with m≥2 and that the penalty parameters $\lambda =\lambda_{i}=\mu_{i}$, i=1,…,n−1 satisfy*

$$c_{1}\sqrt{\Delta^{2-\alpha }\log\Delta }\leq \lambda \leq c_{2}\Delta^{\beta }\delta ,$$

*where $c_{1},c_{2}>0$ are constants, 0<α,β<1, $\delta =o(\Delta^{1-\beta })$, and $\Delta^{2\beta -\max(\alpha ,\beta )}\delta^{2}\to \infty$ when n→∞. The estimates ${\hat{\theta }}_{1},\ldots ,{\hat{\theta }}_{n}$ are obtained by solving the $l_{2}$-GNIO problem *(8)*. Then, under Assumption 1, there exists a constant $c_{3}>0$ such that*

(10) $$\begin{array}{cc} \lim_{n\to \infty }P & \left({\hat{\theta }}_{1}=\cdots ={\hat{\theta }}_{\left\lfloor \tau_{1}-c_{3}\Delta^{\gamma }\right\rfloor }\in \left[{\bar{y}}_{1:q_{1,1}}-\frac{\lambda }{q_{1,1}},{\bar{y}}_{1:q_{1,2}}+\frac{\lambda }{q_{1,2}}\right]\right)=1,\\ \lim_{n\to \infty }P & \left({\hat{\theta }}_{\left\lceil \tau_{i}+c_{3}\Delta^{\gamma }\right\rceil }=\cdots ={\hat{\theta }}_{\left\lfloor \tau_{i+1}-c_{3}\Delta^{\gamma }\right\rfloor }\in \left[{\bar{y}}_{q_{i,3}:q_{i+1,1}}-\frac{\lambda }{q_{i+1,1}-q_{i,3}+1},\right.\right.\\ & \left.\left.{\bar{y}}_{q_{i,3}:q_{i+1,2}}+\frac{\lambda }{q_{i+1,2}-q_{i,3}+1}\right]\right)=1,\;i=1,\ldots ,m-1,\\ \lim_{n\to \infty }P & \left({\hat{\theta }}_{\left\lceil \tau_{m}+c_{3}\Delta^{\gamma }\right\rceil }=\cdots ={\hat{\theta }}_{n}\in \left[{\bar{y}}_{q_{m,3}:n}-\frac{\lambda }{n-q_{m,3}+1},{\bar{y}}_{q_{m,3}:n}+\frac{\lambda }{n-q_{m,3}+1}\right]\right)=1,\\ \lim_{n\to \infty }P & \left({\hat{\theta }}_{\left\lceil \tau_{i}-c_{3}\Delta^{\gamma }\right\rceil }=\cdots ={\hat{\theta }}_{\left\lfloor \tau_{i}+c_{3}\Delta^{\gamma }\right\rfloor }\right)=0,\;i=1,\ldots ,m, \end{array}$$

*where γ=max(α,β), $q_{i,1},q_{i,2}\in \left[\left\lfloor \tau_{i}-c_{3}\Delta^{\gamma }\right\rfloor ,\tau_{i}\right]$, $q_{i,3}\in \left[\tau_{i}+1,\left\lceil \tau_{i}+c_{3}\Delta^{\gamma }\right\rceil \right]$ for i=1,…,m, and ${\bar{y}}_{a:b}=\sum_{i=a}^{b}y_{i}/\left(b-a+1\right)$ for 1≤a≤b≤n. Furthermore, all the interval lengths in (10) are of order O(λ/Δ)=o(1).*

**Remark** **2.** *A common case of Assumption 1 occurs when δ is of constant order and *Δ* is of the same order as n. In this case, requiring the penalty parameters $\lambda =\lambda_{i}=\mu_{i}$, i=1,…,n−1 to satisfy*

$$c_{1}\sqrt{n^{2-\alpha }\log n}\leq \lambda \leq c_{2}n^{\beta },$$

*still ensures that the conclusions of Theorems 2 and 3 hold, where $c_{1},c_{2}$ are constants, 0<α,β<min{1,2β}. Regarding the choice of λ, it is natural to select a minimum value that ensures consistent estimation of change-point locations as larger values reduce the sensitivity to detect changes. Therefore, we recommend using $\lambda =c_{1}\sqrt{n^{1.01}\log n}$. However, in practice, the simpler choice $\lambda =c_{1}\sqrt{n\log n}$ is often sufficient. Furthermore, the constant $c_{1}$ can be selected via the Bayesian Information Criterion (BIC) or simply set to 1.*

Theorem 3 shows that, under certain conditions, the GNIO-DP algorithm achieves consistency in estimating the positions of change-points. All the detected change-points lie within intervals $\left[\left\lceil \tau_{i}-c_{3}\Delta^{\gamma }\right\rceil ,\left\lfloor \tau_{i}+c_{3}\Delta^{\gamma }\right\rfloor \right]$, i=1,…,m, with probability approaching 1. Meanwhile, Theorems 2 and 3 further provide guidance on the selection of parameters $\lambda_{i}$ and $\mu_{i}$. Notably, GNIO-DP operates with linear time complexity of O(n), offering superior computational efficiency compared to other well-known change-point detection algorithms. Nevertheless, GNIO-DP often tends to overestimate, detecting multiple abrupt changes around each true change-point. The proofs of Theorems 2 and 3 are provided in Appendix B.

## 4. Simulation

In this section, we evaluate the performance of the GNIO-DP algorithm on mean-shift models under both single and multiple change-point scenarios. We compare GNIO-DP with four classical change-point detection methods: functional pruning optimal partitioning (FPOP) from [16], pruned exact linear time (PELT) from [14], wild binary segmentation (WBS) from [10], and narrowest-over-threshold (NOT) from [11]. For GNIO-DP, based on Remark 2, we set $\lambda_{i}=\mu_{i}=\sqrt{n\log n}$, whereas, for the other methods, the penalty parameters are used as recommended in their respective original papers.

**Figure 1:**
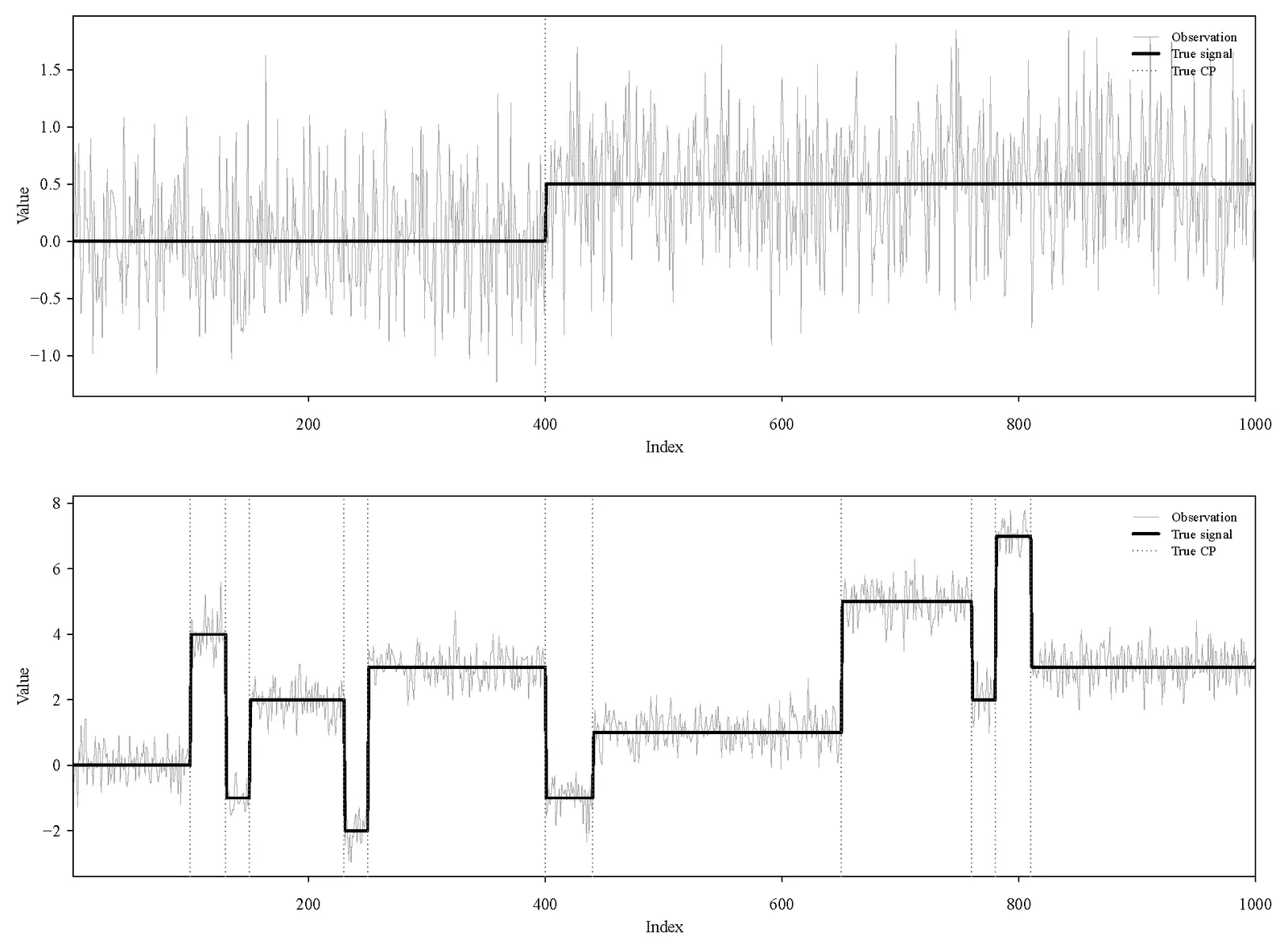
Signal configurations for mean-shift models with single and multiple change-points (n=1000; σ=0.50).

We assess all the methods by the localization error and the runtime in seconds. The following measure

$$\epsilon \left(\hat{M}∥M\right)=\frac{1}{n}\underset{\hat{a}\in \hat{M}}{\sup}\underset{a\in M}{\inf}\left|\hat{a}-a\right|$$

is used in [17] to evaluate the localization error, where M is the true set of change-points and $\hat{M}$ is the estimated set. Figure 1 illustrates the signal configuration for the mean-shift models with single and multiple change-points to be considered in the following analysis. The observations are generated by adding Gaussian white noise to the underlying signal at different signal-to-noise ratios (SNRs), where the SNR is defined as

$$SNR=\frac{\min_{i=1,\ldots ,m}\left|\theta_{\tau_{i}+1}^{*}-\theta_{\tau_{i}}^{*}\right|}{\sigma }.$$

All the experiments are conducted on a 2025 MacBook Air equipped with an Apple M4 chip (8-core CPU; 10-core GPU), 16 GB of unified memory, and a 512 GB SSD, running macOS Sequoia 15.6. All the results are based on 1000 replicates.

### 4.1. Single-Change Setting

The numerical results for the single-change setting are summarized in Table 1 and Table 2. We first consider the results under different SNR levels in Table 1. As expected, the $\epsilon (\hat{M}∥M)$ metric generally increases as the SNR decreases, reflecting the increased difficulty of detecting changes under higher noise levels. GNIO-DP achieves very small estimation errors at SNRs of 10 and 5, with $\epsilon (\hat{M}∥M)$ equal to $3.0\times 10^{-6}$ and $1.4\times 10^{-4}$, respectively. At SNR = 1, the error increases to $7.4\times 10^{-3}$, which remains close to that of NOT and smaller than those of WBS and FPOP. Notably, PELT fails to detect the true change-points when the jump size is 0.5. These results indicate that GNIO-DP maintains competitive change-point estimation accuracy even when the signal becomes relatively weak.

**Figure 2:**
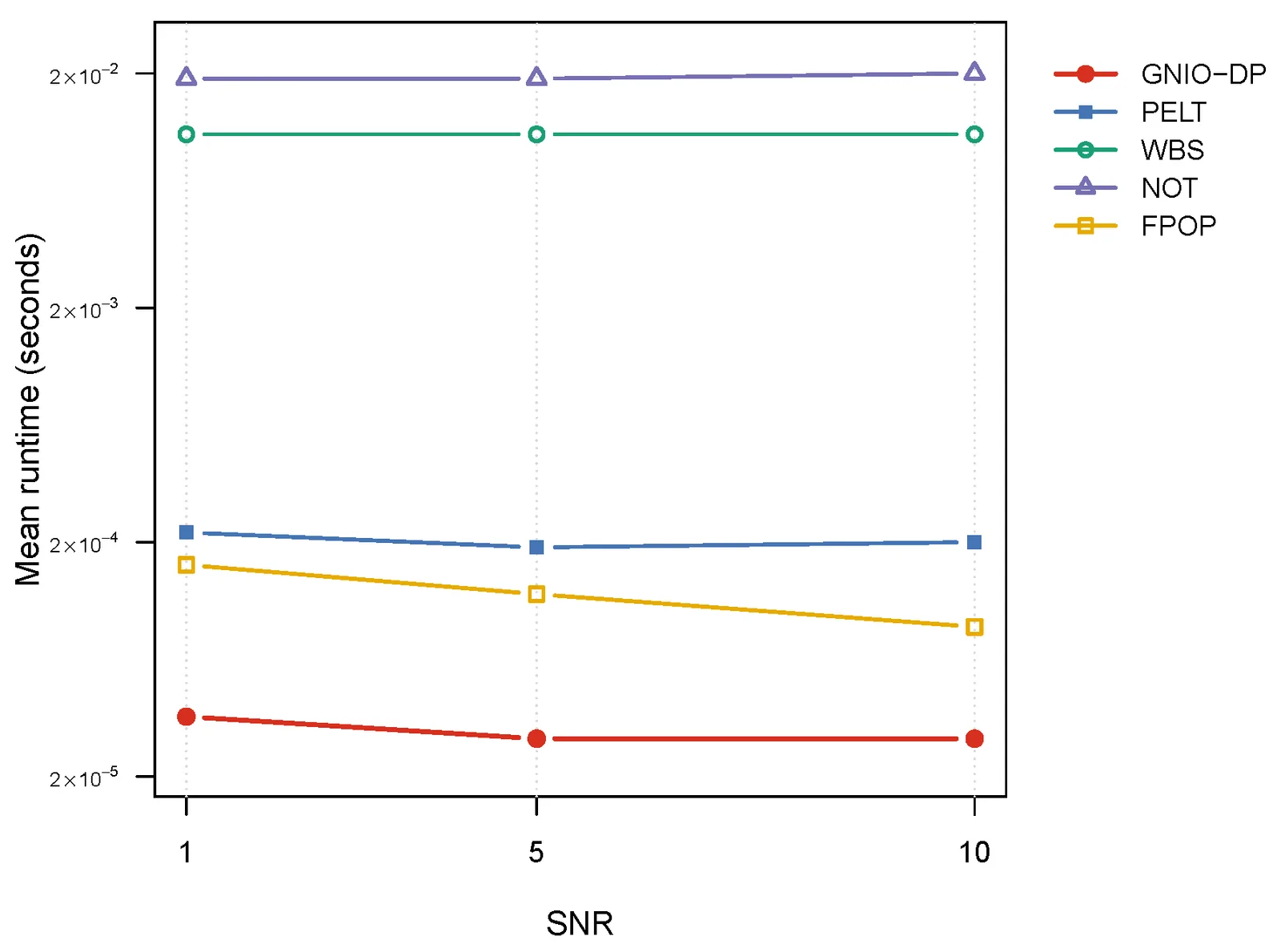
Average runtime with n=1000 and jump size = 0.5 under different SNRs for GNIO-DP and competing methods in the single-change setting.

GNIO-DP exhibits its most pronounced advantage in computational efficiency. As seen from Table 1 and Figure 2, GNIO-DP is consistently the fastest method across all three SNR levels. Its average runtime remains on the order of $10^{-5}$ s, ranging from $2.9\times 10^{-5}$ to $3.6\times 10^{-5}$ s. In comparison, PELT and FPOP require approximately $10^{-4}$ s, while WBS and NOT require approximately $10^{-2}$ s. Thus, GNIO-DP provides substantial computational savings compared with all the competing methods, with the advantage becoming particularly pronounced relative to WBS and NOT. Moreover, its runtime changes only slightly as the SNR decreases, suggesting that its computational efficiency is relatively insensitive to the noise level.

Table 2 further examines the effect of SNR on the $\epsilon (\hat{M}∥M)$ metric when σ=0.5. GNIO-DP exhibits a highly stable estimation error across the three SNR levels, with values of $7.4\times 10^{-3}$, $7.6\times 10^{-3}$, and $7.4\times 10^{-3}$ for SNRs of 10, 5, and 1, respectively. Some competing methods achieve smaller errors under favorable high-SNR conditions. In particular, PELT achieves zero error at SNR = 10 and an error of $6.8\times 10^{-4}$ at SNR = 5. Combined with Table 1, these results indicate that the estimation error of all the methods is influenced not only by the SNR but also by the absolute jump size. When the absolute jump size is sufficiently large, the estimation error tends to stabilize, and PELT can even achieve zero error.

Taken together, Table 1 and Table 2 suggest that GNIO-DP offers a favorable time–accuracy trade-off: although it does not always achieve the smallest localization error, it achieves competitive detection performance with a substantially lower computational cost.

### 4.2. Multiple-Change Setting

**Figure 3:**
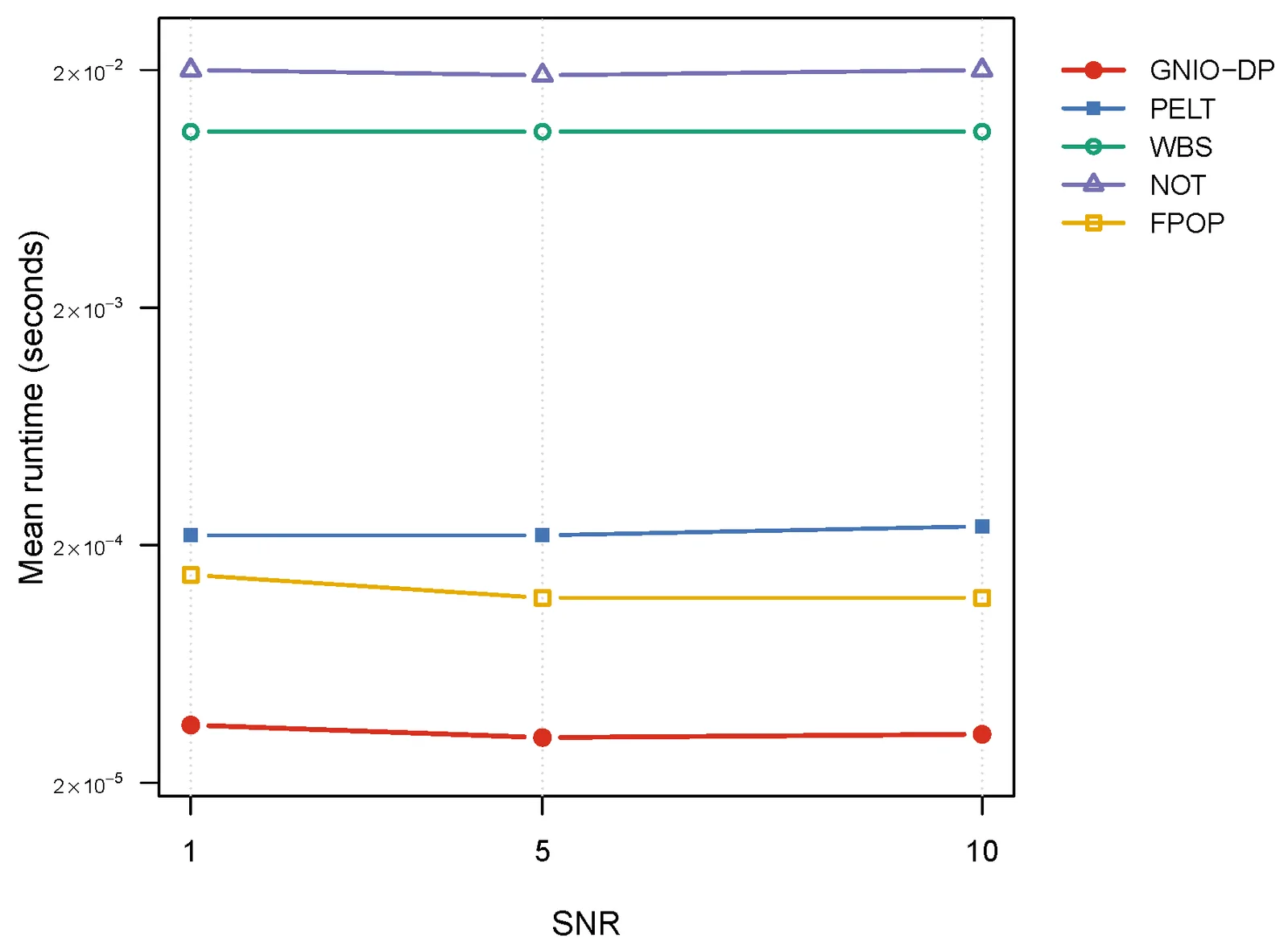
Average runtime with n=1000 under different SNRs for GNIO-DP and competing methods in the multiple-change setting.

**Figure 4:**
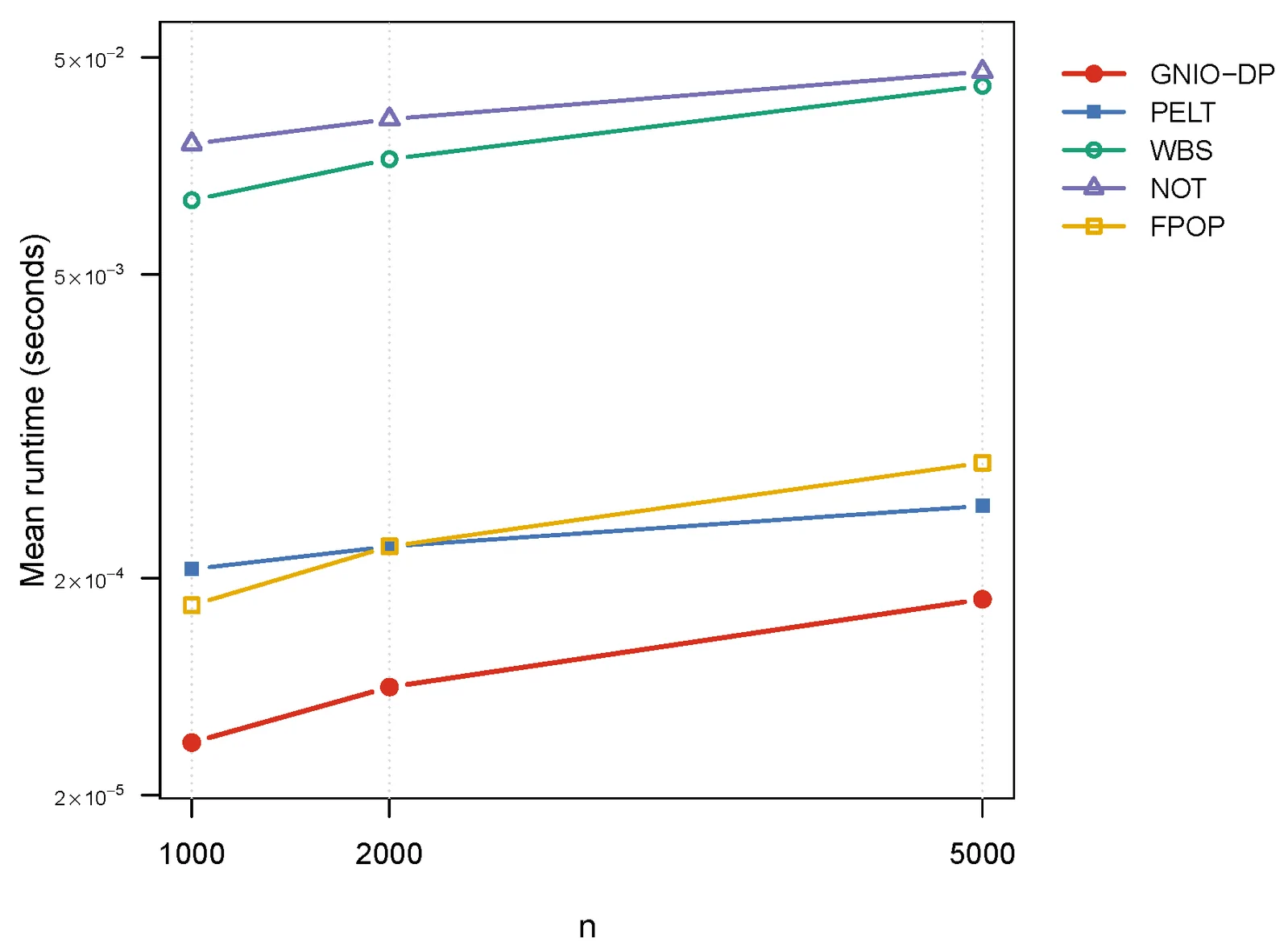
Average runtime with SNR = 1 under different sample sizes for GNIO-DP and competing methods in the multiple-change setting.

Next, we consider the multiple-change setting, where the mean-shift model contains 11 true change-points. The corresponding results are reported in Table 3 and Table 4. As the complexity of the change-point setting increases, the runtime of all the methods correspondingly increases. Moreover, as *n* increases, the performance of all the methods on the $\epsilon (\hat{M}∥M)$ metric improves. Figure 3 and Figure 4 more intuitively demonstrate that, in the multiple-change setting, GNIO-DP consistently achieves the shortest runtime across all three SNR levels and all three sample sizes *n*.

Table 3 compares the performance of the methods under different SNR levels with n=1000. The results further confirm the computational advantage of GNIO-DP observed in the single-change setting. GNIO-DP is consistently the fastest method across all three SNR levels, with average runtimes ranging from $3.1\times 10^{-5}$ to $3.5\times 10^{-5}$ s. Notably, at SNRs of 10 and 5, both GNIO-DP and PELT achieve zero estimation error, while GNIO-DP requires only about one-seventh of the runtime of PELT. This demonstrates that the substantial computational advantage of GNIO-DP does not come at the cost of reduced localization accuracy in these relatively favorable signal-to-noise regimes. Since all the jump sizes exceed 1 in the multiple-change setting, they are sufficiently large that the estimation errors of all the methods remain stable across different SNR levels, which is consistent with the findings in the single-change setting.

We further investigate the effect of the sample size under the challenging low-SNR setting with SNR = 1. The results are presented in Table 4. As *n* increases from 1000 to 5000, GNIO-DP remains the fastest method throughout. Its average runtime increases from $3.5\times 10^{-5}$ s at n=1000 to $1.6\times 10^{-4}$ s at n=5000, whereas the corresponding runtimes of PELT and FPOP increase to $4.3\times 10^{-4}$ and $6.8\times 10^{-4}$ s, respectively. The computational advantage over WBS and NOT is even more pronounced, with their runtimes reaching $3.7\times 10^{-2}$ and $4.3\times 10^{-2}$ s, respectively, at n=5000. Therefore, although the runtime of GNIO-DP naturally increases with the sample size, it remains substantially more computationally efficient than all the competing methods.

Overall, the simulation results demonstrate that GNIO-DP is particularly attractive when computational efficiency is paramount. Although PELT may achieve better performance on the $\epsilon (\hat{M}∥M)$ metric when the jump size exceeds 1, GNIO-DP remains competitive in localization accuracy while clearly outperforming all the competing methods in runtime. Therefore, the proposed method achieves an excellent balance between detection quality and computational cost.

## 5. Real Data Analysis

The well-log data analyzed in [18] were collected from a probe lowered into a borehole. As the probe moves from one type of rock formation to another, the measurements change abruptly. Detecting these changes was the real motivation for collecting this data as it enables rapid identification of formation changes during drilling, allowing timely adjustments to the drill rig setup. Prior to applying change-point detection, the data are standardized by dividing by the overall standard deviation.

**Figure 5:**
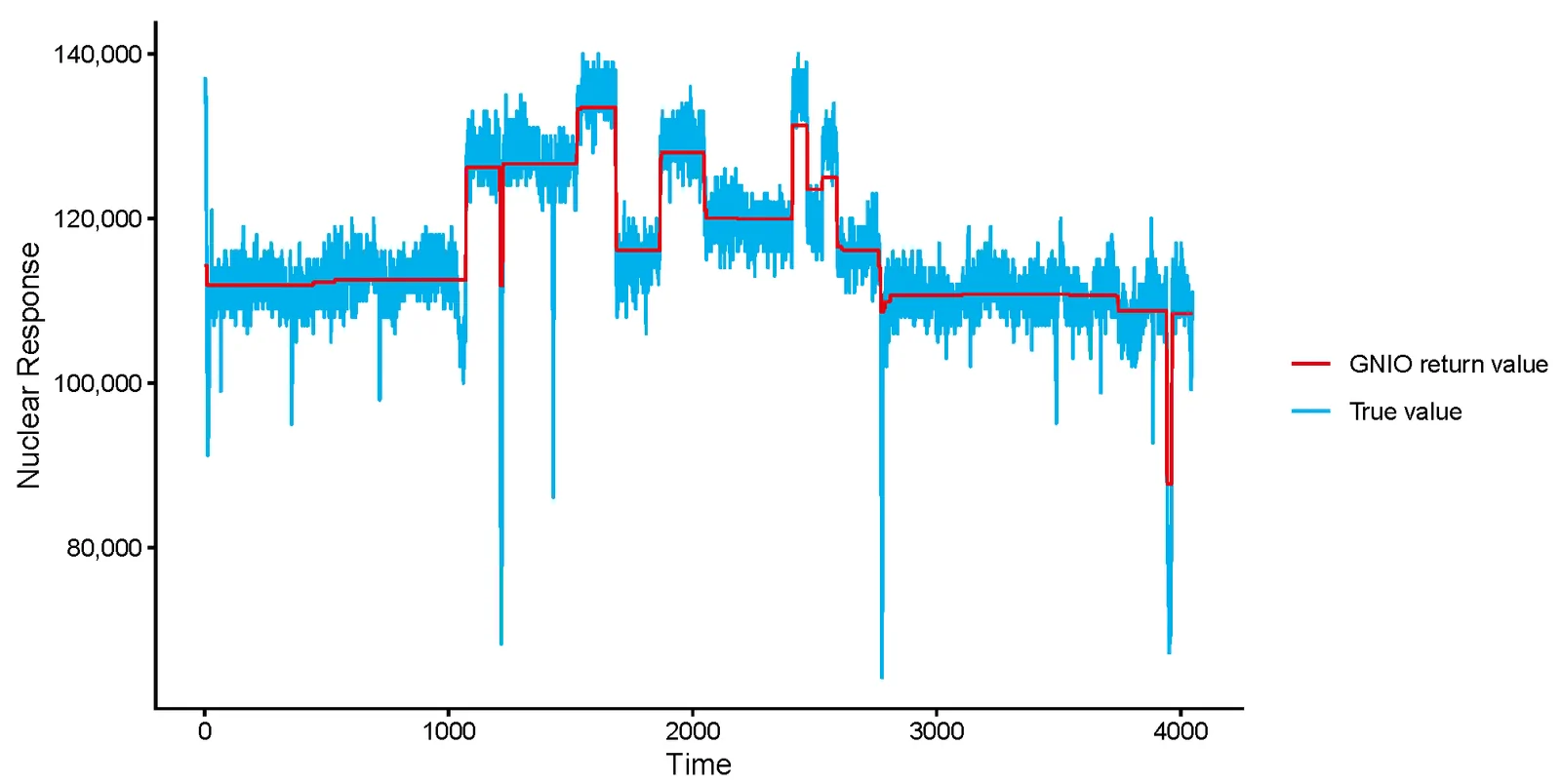
The original well-log data and the estimated result obtained by GNIO-DP.

We use GNIO-DP to analyze this dataset. Here, we set $\lambda_{i}=\mu_{i}=K_{\ell }\sqrt{n\log n}$, where $K_{\ell }=0.05+0.005l$, l=1,…,10, to select the optimal parameters by BIC. The detection results of the GNIO-DP algorithm are shown in Figure 5. The detected significant changes are located at positions 9, 1070, 1211, 1526, 1684, 1866, 2046, 2408, 2469, 2532, 2592, 2767, and 3943. The change-points detected by GNIO-DP are similar to those detected by the method in [18]. Notably, positions 9, 1211, and 3943 are extreme outliers, and our method cannot distinguish between outliers and change-points.

Our method processed the data in $6.4\times 10^{-4}$ s, outperforming the fastest alternative method (FPOP, $7.6\times 10^{-4}$ s). Overall, our method excels in both computational efficiency and quality of estimation, yielding highly competitive results that are well suited for practical applications.

## 6. Conclusions

In this paper, we adapt the GNIO-DP algorithm for change-point detection through appropriate parameter selection, thereby obtaining a change-point detection method with O(n) computational complexity. At the same time, we provide the theoretical properties of the proposed change-point detection method and prove the reliability and effectiveness of the GNIO-DP algorithm for this task. The simulation results demonstrate that our method exhibits strong change-point detection capabilities. Compared with traditional methods, our method is the fastest in most cases. Our method is also applied to well-log data with satisfactory results. In summary, our method realizes a favorable time–accuracy trade-off: it significantly reduces computation time without materially sacrificing the practical quality of detection. This work also suggests several promising directions for future research, including extending GNIO-DP to high-dimensional settings, relaxing the i.i.d. assumption to accommodate dependent errors under suitable weak-dependence or mixing conditions, developing online versions for streaming data, and distinguishing persistent structural changes from transient anomalies.

## Figures and Tables

**Table 1:** Simulation results for the single-change mean-shift model with n=1000 and jump size = 0.5 under different SNRs.

Method	Metric	SNR = 10	SNR = 5	SNR = 1
GNIO-DP	$\epsilon (\hat{M}∥M)$	$3.0\times 10_{\left(5.5\times 10^{-5}\right)}^{-6}$	$1.4\times 10_{\left(4.2\times 10^{-4}\right)}^{-4}$	$7.4\times 10_{\left(9.8\times 10^{-3}\right)}^{-3}$
	Average Runtime (s)	$2.9\times 10^{-5}$	$2.9\times 10^{-5}$	$3.6\times 10^{-5}$
PELT	$\epsilon (\hat{M}∥M)$	${\mathrm{NA}}_{\left(\mathrm{NA}\right)}$	${\mathrm{NA}}_{\left(\mathrm{NA}\right)}$	${\mathrm{NA}}_{\left(\mathrm{NA}\right)}$
	Average Runtime (s)	$2.0\times 10^{-4}$	$1.9\times 10^{-4}$	$2.2\times 10^{-4}$
WBS	$\epsilon (\hat{M}∥M)$	$1.1\times 10_{\left(6.4\times 10^{-2}\right)}^{-2}$	$8.4\times 10_{\left(5.8\times 10^{-2}\right)}^{-3}$	$1.5\times 10_{\left(6.7\times 10^{-2}\right)}^{-2}$
	Average Runtime (s)	$1.1\times 10^{-2}$	$1.1\times 10^{-2}$	$1.1\times 10^{-2}$
NOT	$\epsilon (\hat{M}∥M)$	$4.1\times 10_{\left(3.8\times 10^{-2}\right)}^{-3}$	$5.3\times 10_{\left(4.6\times 10^{-2}\right)}^{-3}$	$7.2\times 10_{\left(4.2\times 10^{-2}\right)}^{-3}$
	Average Runtime (s)	$2.0\times 10^{-2}$	$1.9\times 10^{-2}$	$1.9\times 10^{-2}$
FPOP	$\epsilon (\hat{M}∥M)$	$6.4\times 10_{\left(2.0\times 10^{-3}\right)}^{-2}$	$6.4\times 10_{\left(1.1\times 10^{-2}\right)}^{-2}$	$1.1\times 10_{\left(1.1\times 10^{-1}\right)}^{-1}$
	Average Runtime (s)	$8.7\times 10^{-5}$	$1.2\times 10^{-4}$	$1.6\times 10^{-4}$

**Table 2:** Simulation results for the single-change mean-shift model with n=1000 and σ=0.5 under different SNRs.

Method	Metric	SNR = 10	SNR = 5	SNR = 1
GNIO-DP	$\epsilon (\hat{M}∥M)$	$7.4\times 10_{\left(1.0\times 10^{-2}\right)}^{-3}$	$7.6\times 10_{\left(1.0\times 10^{-2}\right)}^{-3}$	$7.4\times 10_{\left(9.8\times 10^{-3}\right)}^{-3}$
PELT	$\epsilon (\hat{M}∥M)$	$0.0000_{\left(0.0000\right)}$	$6.8\times 10_{\left(1.2\times 10^{-3}\right)}^{-4}$	${\mathrm{NA}}_{\left(\mathrm{NA}\right)}$
WBS	$\epsilon (\hat{M}∥M)$	$9.2\times 10_{\left(5.8\times 10^{-2}\right)}^{-3}$	$1.2\times 10_{\left(6.6\times 10^{-2}\right)}^{-2}$	$1.5\times 10_{\left(6.7\times 10^{-2}\right)}^{-2}$
NOT	$\epsilon (\hat{M}∥M)$	$4.7\times 10_{\left(4.3\times 10^{-2}\right)}^{-3}$	$5.8\times 10_{\left(4.7\times 10^{-2}\right)}^{-3}$	$7.2\times 10_{\left(4.2\times 10^{-2}\right)}^{-3}$
FPOP	$\epsilon (\hat{M}∥M)$	$3.2\times 10_{\left(4.7\times 10^{-4}\right)}^{-4}$	$3.3\times 10_{\left(7.8\times 10^{-2}\right)}^{-2}$	$1.1\times 10_{\left(1.1\times 10^{-1}\right)}^{-1}$

**Table 3:** Simulation results for the multiple-change mean-shift model with n=1000 under different SNRs. The true number of change-points is 11.

Method	Metric	SNR = 10	SNR = 5	SNR = 1
GNIO-DP	$\epsilon (\hat{M}∥M)$	$0.0000_{\left(0.0000\right)}$	$0.0000_{\left(0.0000\right)}$	$1.2\times 10_{\left(1.8\times 10^{-3}\right)}^{-3}$
	Average Runtime (s)	$3.2\times 10^{-5}$	$3.1\times 10^{-5}$	$3.5\times 10^{-5}$
PELT	$\epsilon (\hat{M}∥M)$	$0.0000_{\left(0.0000\right)}$	$0.0000_{\left(0.0000\right)}$	$5.4\times 10_{\left(2.3\times 10^{-4}\right)}^{-5}$
	Average Runtime (s)	$2.4\times 10^{-4}$	$2.2\times 10^{-4}$	$2.2\times 10^{-4}$
WBS	$\epsilon (\hat{M}∥M)$	$1.3\times 10_{\left(1.2\times 10^{-2}\right)}^{-3}$	$6.5\times 10_{\left(6.7\times 10^{-3}\right)}^{-4}$	$1.9\times 10_{\left(1.3\times 10^{-2}\right)}^{-3}$
	Average Runtime (s)	$1.1\times 10^{-2}$	$1.1\times 10^{-2}$	$1.1\times 10^{-2}$
NOT	$\epsilon (\hat{M}∥M)$	$1.5\times 10_{\left(1.2\times 10^{-2}\right)}^{-3}$	$1.2\times 10_{\left(9.8\times 10^{-3}\right)}^{-3}$	$2.1\times 10_{\left(1.2\times 10^{-2}\right)}^{-3}$
	Average Runtime (s)	$2.0\times 10^{-2}$	$1.9\times 10^{-2}$	$2.0\times 10^{-2}$
FPOP	$\epsilon (\hat{M}∥M)$	$3.0\times 10_{\left(1.6\times 10^{-4}\right)}^{-3}$	$3.2\times 10_{\left(3.7\times 10^{-4}\right)}^{-3}$	$4.3\times 10_{\left(6.9\times 10^{-3}\right)}^{-3}$
	Average Runtime (s)	$1.2\times 10^{-4}$	$1.2\times 10^{-4}$	$1.5\times 10^{-4}$

**Table 4:** Simulation results for the multiple-change mean-shift model with SNR = 1 under different *n*. The true number of change-points is 11.

Method	Metric	n = 1000	n = 2000	n = 5000
GNIO-DP	$\epsilon (\hat{M}∥M)$	$1.2\times 10_{\left(1.8\times 10^{-3}\right)}^{-3}$	$2.9\times 10_{\left(6.0\times 10^{-4}\right)}^{-4}$	$2.2\times 10_{\left(1.2\times 10^{-3}\right)}^{-4}$
	Average Runtime (s)	$3.5\times 10^{-5}$	$6.3\times 10^{-5}$	$1.6\times 10^{-4}$
PELT	$\epsilon (\hat{M}∥M)$	$5.4\times 10_{\left(2.3\times 10^{-4}\right)}^{-5}$	$5.1\times 10_{\left(2.2\times 10^{-4}\right)}^{-5}$	$4.4\times 10_{\left(2.2\times 10^{-4}\right)}^{-5}$
	Average Runtime (s)	$2.2\times 10^{-4}$	$2.8\times 10^{-4}$	$4.3\times 10^{-4}$
WBS	$\epsilon (\hat{M}∥M)$	$1.9\times 10_{\left(1.3\times 10^{-2}\right)}^{-3}$	$7.3\times 10_{\left(9.3\times 10^{-3}\right)}^{-4}$	$5.5\times 10_{\left(1.5\times 10^{-2}\right)}^{-4}$
	Average Runtime (s)	$1.1\times 10^{-2}$	$1.7\times 10^{-2}$	$3.7\times 10^{-2}$
NOT	$\epsilon (\hat{M}∥M)$	$2.1\times 10_{\left(1.2\times 10^{-2}\right)}^{-3}$	$1.5\times 10_{\left(1.3\times 10^{-3}\right)}^{-3}$	$2.6\times 10_{\left(3.8\times 10^{-2}\right)}^{-3}$
	Average Runtime (s)	$2.0\times 10^{-2}$	$2.6\times 10^{-2}$	$4.3\times 10^{-2}$
FPOP	$\epsilon (\hat{M}∥M)$	$4.3\times 10_{\left(6.9\times 10^{-3}\right)}^{-3}$	$4.2\times 10_{\left(7.7\times 10^{-3}\right)}^{-3}$	$1.3\times 10_{\left(4.5\times 10^{-3}\right)}^{-3}$
	Average Runtime (s)	$1.5\times 10^{-4}$	$2.8\times 10^{-4}$	$6.8\times 10^{-4}$

## Data Availability

The data used in our paper can be found via the link https://github.com/alan-turing-institute/TCPD/tree/master/datasets/well_log (accessed on 5 January 2026), which is publicly accessible.

## Abbreviations

The following abbreviations are used in this manuscript:

GNIO	Generalized Nearly Isotonic Optimization
DP	Dynamic Programming
PELT	Pruned Exact Linear Time
WBS	Wild Binary Segmentation
NOT	Narrowest-Over-Threshold
FPOP	Functional Pruning Optimal Partitioning

## Appendix A. A Detailed Discussion of the GNIO-DP Algorithm

### Appendix A.1. A Simple Illustrative Example

To illustrate the key steps of the GNIO-DP algorithm, consider the following simple example with n=2. Let

$$\left(y_{1},y_{2}\right)=\left(2,2.4\right),$$

and consider the following GNIO problem

$$\min_{\theta \in R^{2}}\left\{\sum_{i=1}^{2}0.5{\left(\theta_{i}-y_{i}\right)}^{2}+\lambda_{1}{\left(\theta_{1}-\theta_{2}\right)}_{+}+\mu_{1}{\left(\theta_{2}-\theta_{1}\right)}_{+}\right\},$$

where $\lambda_{1}=3$ and $\mu_{1}=0.5$.

We first initialize $h_{1}\left(\theta_{1}\right)=0$, which gives

$$g_{1}\left(\theta_{1}\right)=h_{1}\left(\theta_{1}\right)+f_{1}\left(\theta_{1}\right)=0.5{\left(\theta_{1}-y_{1}\right)}^{2}=0.5{\left(\theta_{1}-2\right)}^{2}.$$

The two breakpoints are given by $b_{2}^{-}=\arg\min_{z\in R}\left(g_{1}\left(z\right)+\lambda_{1}z\right)$ and $b_{2}^{+}=\arg\min_{z\in R}\left(g_{1}\left(z\right)-\mu_{1}z\right)$, yielding

$$b_{2}^{-}=-1,\;b_{2}^{+}=2.5.$$

By (6), we obtain

$$\begin{array}{cc} \; & h_{2}\left(\theta_{2}\right)=\left\{\begin{array}{c} 0.5{\left(-1-2\right)}^{2}+3\left(-1-\theta_{2}\right),\\ 0.5{\left(\theta_{2}-2\right)}^{2},\\ 0.5{\left(2.5-2\right)}^{2}+0.5\left(\theta_{2}-2.5\right), \end{array}\right.=\left\{\begin{array}{cc} -3\theta_{2}+1.5, & \theta_{2}<-1,\\ 0.5{\left(\theta_{2}-2\right)}^{2}, & -1\leq \theta_{2}\leq 2.5,\\ 0.5\theta_{2}-1.125, & \theta_{2}>2.5, \end{array}\right.\\ \; & g_{2}\left(\theta_{2}\right)=h_{2}\left(\theta_{2}\right)+f_{2}\left(\theta_{2}\right)=\left\{\begin{array}{cc} 0.5\theta_{2}^{2}-5.4\theta_{2}+4.38, & \theta_{2}<-1,\\ \theta_{2}^{2}-4.4\theta_{2}+4.88, & -1\leq \theta_{2}\leq 2.5,\\ 0.5\theta_{2}^{2}-1.9\theta_{2}+1.755, & \theta_{2}>2.5. \end{array}\right. \end{array}$$

Then, the minimizer of $g_{2}\left(\theta_{2}\right)$ is

$${\hat{\theta }}_{2}=\arg\min_{\theta_{2}\in R}g_{2}\left(\theta_{2}\right)=2.2.$$

Finally, the optimal value of $\theta_{1}$ is recovered by (7); that is,

$${\hat{\theta }}_{1}=\min\left(b_{2}^{+},\max\left(b_{2}^{-},\hat{\theta_{2}}\right)\right)=\min\left(2.5,\max\left(-1,2.2\right)\right)=2.2.$$

Hence, the optimal solution is $\left({\hat{\theta }}_{1},{\hat{\theta }}_{2}\right)=\left(2.2,2.2\right)$.

### Appendix A.2. Piecewise Quadratic Structure Under Quadratic Loss

If

(A1) $$f_{i}\left(\theta_{i}\right)=f\left(\theta_{i},y_{i}\right)=\frac{1}{2}{\left(\theta_{i}-y_{i}\right)}^{2},$$

the functions $h_{i}$ and $g_{i}$ inherit a particularly simple structure. Proposition 4 of Yu et al. [15] shows that, under (A1), all $h_{i}$ and $g_{i}$ in the recursion of the GNIO-DP algorithm are univariate convex differentiable piecewise quadratic functions, and their derivatives cover the full real line:

(A2) $$\underset{\theta_{i}\in R}{\inf}g_{i}^{\prime }\left(\theta_{i}\right)=-\infty ,\;\underset{\theta_{i}\in R}{\sup}g_{i}^{\prime }\left(\theta_{i}\right)=+\infty ,\;i=1,\ldots ,n.$$

Let h:R→R be a univariate convex differentiable piecewise quadratic function with breakpoints

$$\beta_{1}<\beta_{2}<\cdots <\beta_{K_{h}}.$$

These breakpoints partition R into $K_{h}+1$ intervals,

$$\left(-\infty ,\beta_{1}\right),\;\left[\beta_{\ell },\beta_{\ell +1}\right),\;\ell =1,\ldots ,K_{h}-1,\;\left[\beta_{K_{h}},+\infty \right),$$

on each of which *h* takes the form of a quadratic polynomial

$$h\left(x\right)=a_{j}x^{2}+b_{j}x+c_{j},\;x\in I_{j},\;j=1,\ldots ,K_{h}+1,$$

where $a_{j}\geq 0$. In the implementation of GNIO-DP, it is convenient to ignore the intercepts $c_{j}$ and to store *h* via the following difference-of-coefficients representation:

$$h\equiv [B^{h},\;D^{h},\;c_{L}^{h},\;c_{R}^{h},\;K_{h}],$$

where

- $B^{h}:=\left\{\beta_{1},\ldots ,\beta_{K_{h}}\right\}$ is the sorted list of breakpoints;
- $D^{h}$ is a list of length $K_{h}$ whose *ℓ*-th entry stores the difference between the coefficients of the (ℓ+1)-th and *ℓ*-th pieces
  $$d_{\ell }:=\left(a_{\ell +1}-a_{\ell },\;b_{\ell +1}-b_{\ell }\right),\;\ell =1,\ldots ,K_{h};$$
- $c_{L}^{h}:=\left(a_{1},b_{1}\right)$ and $c_{R}^{h}:=\left(a_{K_{h}+1},b_{K_{h}+1}\right)$ store the coefficients of the leftmost and rightmost quadratic pieces;
- $K_{h}$ is the number of breakpoints.

With this notation, the quadratic loss (A1) can be written as

$$f_{i}=\left[\emptyset ,\;\emptyset ,\;\left(\frac{1}{2},-y_{i}\right),\;\left(\frac{1}{2},-y_{i}\right),\;0\right],\;i=1,\ldots ,n.$$

Hence, if $h_{i}$ has representation $h_{i}=\left[B^{h_{i}},D^{h_{i}},c_{L}^{h_{i}},c_{R}^{h_{i}},K_{h_{i}}\right]$, then

(A3) $$g_{i}=\left[B^{h_{i}},\;D^{h_{i}},\;c_{L}^{h_{i}}+\left(\frac{1}{2},-y_{i}\right),\;c_{R}^{h_{i}}+\left(\frac{1}{2},-y_{i}\right),\;K_{h_{i}}\right],$$

i.e., $g_{i}$ is obtained by simply adding $\left(\frac{1}{2},-y_{i}\right)$ to the coefficients of the leftmost and rightmost pieces of $h_{i}$ without modifying the breakpoint set $B^{h_{i}}$ and the difference list $D^{h_{i}}$. The operation $h_{i}\mapsto g_{i}$ has O(1) cost.

Moreover, by (A2) and the piecewise quadratic structure, the breakpoints $\left(b_{i}^{-},b_{i}^{+}\right)$ in (6) can be characterized by

$$g_{i-1}^{\prime }\left(b_{i}^{-}\right)=-\lambda_{i-1},\;g_{i-1}^{\prime }\left(b_{i}^{+}\right)=\mu_{i-1},\;i=2,\ldots ,n.$$

On each interval $I_{j}$, we have $g_{i-1}^{\prime }\left(x\right)=2a_{j}x+b_{j}$. Since $g_{i-1}^{\prime }$ is globally nondecreasing, $b_{i}^{-}$ and $b_{i}^{+}$ can be found by scanning the pieces of $g_{i-1}$ from left to right and from right to left, respectively, and solving simple linear equations on the “correct” pieces. During these scans, entire pieces whose endpoint derivatives fall strictly below $-\lambda_{i-1}$ or strictly above $\mu_{i-1}$ can be discarded, which is crucial for the efficiency of GNIO-DP.

### Appendix A.3. The Derivation of O(n) Complexity

The structural properties established above help to explain the high efficiency of the GNIO-DP algorithm in quadratic GNIO problems, resulting in linear runtime in practice.

First, if $g_{i-1}$ is convex and piecewise quadratic, the truncation step (6) produces $h_{i}$ of the same form. Consequently, the update $g_{i}=f_{i}+h_{i}$ can be carried out efficiently using the compact representation (A3).

Second, new breakpoints arise only from solving for the roots of the (monotone) derivative $g_{i-1}^{\prime }$. Proposition 4 of Yu et al. [15] states that $g_{i-1}^{\prime }$ spans (−∞,+∞), which implies that each truncation introduces at most two new breakpoints.

Third, the total number of pieces processed remains O(n), yielding an amortized constant cost per update.

Finally, once the breakpoint intervals ${\left\{\left(b_{i}^{-},b_{i}^{+}\right)\right\}}_{i=2}^{n}$ are stored, the optimal solution $\hat{\theta }$ can be recovered via the closed-form backward recursion (7).

Combining these observations, one can show that an implementation of GNIO-DP for the quadratic GNIO problem runs in O(n) time. This efficiency is achieved under mild assumptions on the regularization parameters $0\leq \lambda_{i},\mu_{i}\leq +\infty$.

## Appendix B. The Proofs of Theorems 2 and 3

### Appendix B.1. The Proof of Theorem 2

In the following, we use $c_{4},c_{5},c_{6},\ldots$ to denote different positive constants.

As shown in Section 2.2.2, we have

(A4) $${\hat{\theta }}_{i-1}=\min\left(b_{i}^{+},\max\left(b_{i}^{-},{\hat{\theta }}_{i}\right)\right),$$

where $b_{i}^{-},b_{i}^{+}$ satisfy

(A5) $$g_{i-1}^{\prime }\left(b_{i}^{-}\right)=-\lambda_{i-1}\;\;\mathrm{and}\;\;g_{i-1}^{\prime }\left(b_{i}^{+}\right)=\mu_{i-1},$$

and $g_{i-1}$ is a piecewise quadratic function defined in [15] as follows. Define $h_{1}\left(\theta_{1}\right)=0$ for i=2,…,n,

(A6) $$\begin{array}{cc} \; & g_{i-1}\left(\theta_{i-1}\right)=h_{i-1}\left(\theta_{i-1}\right)+f_{i-1}\left(\theta_{i-1}\right),\\ \; & h_{i}\left(\theta_{i}\right)=\left\{\begin{array}{cc} g_{i-1}\left(b_{i}^{-}\right)+\lambda_{i-1}\left(b_{i}^{-}-\theta_{i}\right) & \;\mathrm{if}\;\theta_{i}<b_{i}^{-},\\ g_{i-1}\left(\theta_{i}\right) & \;\mathrm{if}\;b_{i}^{-}\leq \theta_{i}\leq b_{i}^{+},\\ g_{i-1}\left(b_{i}^{+}\right)+\mu_{i-1}\left(\theta_{i}-b_{i}^{+}\right) & \;\mathrm{if}\;\theta_{i}>b_{i}^{+}. \end{array}\right. \end{array}$$

where $f_{i}\left(\theta_{i}\right)=\frac{1}{2}{\left(\theta_{i}-y_{i}\right)}^{2}$, i=1,…,n.

Based on (A4), we know that, for any i=2,…,n,

$${\hat{\theta }}_{i-1}={\hat{\theta }}_{i}\;\;\mathrm{if}\;\mathrm{and}\;\mathrm{only}\;\mathrm{if}\;\;b_{i}^{-}\leq {\hat{\theta }}_{i}\leq b_{i}^{+}.$$

Therefore, we are going to analyze $b_{i}^{-},b_{i}^{+}$, i=2,…,n to demonstrate (9). Once $g_{i-1}^{\prime }$ is known, $b_{i}^{-},b_{i}^{+}$ can be computed via (A5).

First, we start a simple recursion from i=1. Based on (A6), it is easy to see that $g_{1}^{\prime }\left(x\right)=x-y_{1}$, and then

$$b_{2}^{-}=y_{1}-\lambda \;\;\mathrm{and}\;\;b_{2}^{+}=y_{1}+\lambda .$$

Similarly, we can get

$$\begin{array}{cc} \; & g_{2}^{\prime }\left(x\right)=\left\{\begin{array}{cc} x-y_{2}-\lambda & \;\mathrm{if}\;x<y_{1}-\lambda ,\\ 2x-y_{1}-y_{2} & \;\mathrm{if}\;y_{1}-\lambda \leq x\leq y_{1}+\lambda ,\\ x-y_{2}+\lambda & \;\mathrm{if}\;x>y_{1}+\lambda . \end{array}\right. \end{array}$$

The values of $y_{1}$ and $y_{2}$ determine the results of $b_{3}^{-}$ and $b_{3}^{+}$.

(i) When $y_{2}<y_{1}-3\lambda$, we have $b_{3}^{-}=y_{2}$, $b_{3}^{+}=y_{2}+2\lambda$, and then

$$\begin{array}{cc} \; & g_{3}^{\prime }\left(x\right)=\left\{\begin{array}{cc} x-y_{3}-\lambda & \;\mathrm{if}\;x<y_{2},\\ 2x-y_{2}-y_{3}-\lambda & \;\mathrm{if}\;y_{2}\leq x\leq y_{2}+2\lambda ,\\ x-y_{3}+\lambda & \;\mathrm{if}\;x>y_{2}+2\lambda . \end{array}\right. \end{array}$$

(ii) When $y_{1}-3\lambda \leq y_{2}\leq y_{1}-\lambda$, we have $b_{3}^{-}=y_{2}$, $b_{3}^{+}=\left(y_{1}+y_{2}+\lambda \right)/2$, and then

$$\begin{array}{cc} \; & g_{3}^{\prime }\left(x\right)=\left\{\begin{array}{cc} x-y_{3}-\lambda & \;\mathrm{if}\;x<y_{2},\\ 2x-y_{2}-y_{3}-\lambda & \;\mathrm{if}\;y_{2}\leq x\leq y_{1}-\lambda ,\\ 3x-y_{1}-y_{2}-y_{3} & \;\mathrm{if}\;y_{1}-\lambda <x\leq \left(y_{1}+y_{2}+\lambda \right)/2,\\ x-y_{3}+\lambda & \;\mathrm{if}\;x>(y_{1}+y_{2}+\lambda )/2. \end{array}\right. \end{array}$$

(iii) When $y_{1}-\lambda <y_{2}<y_{1}+\lambda$, we have $b_{3}^{-}=\left(y_{1}+y_{2}-\lambda \right)/2$, $b_{3}^{+}=\left(y_{1}+y_{2}+\lambda \right)/2$, and then

$$\begin{array}{cc} \; & g_{3}^{\prime }\left(x\right)=\left\{\begin{array}{cc} x-y_{3}-\lambda & \;\mathrm{if}\;x<(y_{1}+y_{2}-\lambda )/2,\\ 3x-y_{1}-y_{2}-y_{3} & \;\mathrm{if}\;\left(y_{1}+y_{2}-\lambda \right)/2\leq x\leq \left(y_{1}+y_{2}+\lambda \right)/2,\\ x-y_{3}+\lambda & \;\mathrm{if}\;x>(y_{1}+y_{2}+\lambda )/2. \end{array}\right. \end{array}$$

(iv) When $y_{1}+\lambda \leq y_{2}\leq y_{1}+3\lambda$, we have $b_{3}^{-}=\left(y_{1}+y_{2}-\lambda \right)/2$, $b_{3}^{+}=y_{2}$, and then

$$\begin{array}{cc} \; & g_{3}^{\prime }\left(x\right)=\left\{\begin{array}{cc} x-y_{3}-\lambda & \;\mathrm{if}\;x<(y_{1}+y_{2}-\lambda )/2,\\ 3x-y_{1}-y_{2}-y_{3} & \;\mathrm{if}\;\left(y_{1}+y_{2}-\lambda \right)/2\leq x\leq y_{1}+\lambda ,\\ 2x-y_{2}-y_{3}+\lambda & \;\mathrm{if}\;y_{1}+\lambda <x\leq y_{2},\\ x-y_{3}+\lambda & \;\mathrm{if}\;x>y_{2}. \end{array}\right. \end{array}$$

(v) When $y_{2}>y_{1}+3\lambda$, we have $b_{3}^{-}=y_{2}-2\lambda$, $b_{3}^{+}=y_{2}$, and then

$$\begin{array}{cc} \; & g_{3}^{\prime }\left(x\right)=\left\{\begin{array}{cc} x-y_{3}-\lambda & \;\mathrm{if}\;x<y_{2}-2\lambda ,\\ 2x-y_{2}-y_{3}+\lambda & \;\mathrm{if}\;y_{2}-2\lambda \leq x\leq y_{2},\\ x-y_{3}+\lambda & \;\mathrm{if}\;x>y_{2}. \end{array}\right. \end{array}$$

It can be observed that, in scenarios (i) and (v), $b_{3}^{-}$, $b_{3}^{+}$, and $g_{3}^{\prime }\left(x\right)$ no longer contain information regarding $y_{1}$. In the following, Step 1 and Step 3 ensure that scenarios analogous to (i) and (v) are absent when there is no change-point but emerge near a change-point. Meanwhile, Step 2 ensures that, when no change-point exists, the information of $y_{1},\ldots ,y_{i-1}$ will be stored in numerous $b_{i}^{-}$ and $b_{i}^{+}$, similar to what occurs in (ii), (iii), and (iv).

For simplicity, we henceforth assume that all the interval boundaries are integers by default. In practice, this can be achieved by rounding them up or down as needed.

Step 1: Prove that, when $\left[a,a+c_{4}\Delta^{\alpha }\right]\subset \left[1,\tau_{1}\right]$,

(A7) $$\lim_{n\to \infty }P\left(\overset{a+c_{4}\Delta^{\alpha }}{\underset{b=a}{⋂}}\left\{\left|\frac{b}{a}\sum_{i=1}^{a}y_{i}-\sum_{i=1}^{b}y_{i}\right|<\frac{a+b}{a}\lambda \right\}\right)=1.$$

From (A7), we can derive

$$\lim_{n\to \infty }P\left(\overset{a+c_{4}\Delta^{\alpha }}{\underset{b=a}{⋂}}\left\{\frac{\sum_{i=1}^{a}y_{i}-\lambda }{a}<\frac{\sum_{i=1}^{b}y_{i}+\lambda }{b}\right\}\right)=1,$$

and

$$\lim_{n\to \infty }P\left(\overset{a+c_{4}\Delta^{\alpha }}{\underset{b=a}{⋂}}\left\{\frac{\sum_{i=1}^{a}y_{i}+\lambda }{a}>\frac{\sum_{i=1}^{b}y_{i}-\lambda }{b}\right\}\right)=1,$$

ensuring that scenario (i) does not occur in interval $\left[i,i+c_{4}\Delta^{\alpha }\right]\subset \left[1,\tau_{1}\right]$ when $b_{i}^{-}=\left(\sum_{j=1}^{i-1}y_{j}-\lambda \right)/\left(i-1\right)$ and similarly preventing scenario (v) in interval $\left[i,i+c_{4}\Delta^{\alpha }\right]\subset \left[1,\tau_{1}\right]$ when $b_{i}^{+}=\left(\sum_{j=1}^{i-1}y_{j}+\lambda \right)/\left(i-1\right)$.

Denote $A_{a,b}=\frac{b}{a}\sum_{i=1}^{a}y_{i}-\sum_{i=1}^{b}y_{i}$ for a<b. It can be seen that $A_{a,b}$ is a linear combination of independent sub-Gaussian random variables $\left\{y_{1},\ldots ,y_{b}\right\}$, so $A_{a,b}$ is a sub-Gaussian random variable with

$${∥A_{a,b}∥}_{\psi_{2}}\leq \sigma \sqrt{{\left(\frac{b}{a}-1\right)}^{2}a+\left(b-a\right)}=\sigma \sqrt{\frac{b(b-a)}{a}}$$

for a<b. And $E(A_{a,b})=0$ when there is no change-point between *a* and *b*.

According to the tail probability inequality for the sub-Gaussian distribution, there exists an absolute constant $c_{5}>0$ such that, for any t>0, we have

(A8) $$P\left(\left|A_{a,b}\right|>t\right)\leq 2\exp\left(-\frac{c_{5}t^{2}}{{∥A_{a,b}∥}_{\psi_{2}}^{2}}\right).$$

Therefore, as n→∞,

$$\begin{array}{cc} P\left(\overset{a+c_{4}\Delta^{\alpha }}{\underset{b=a}{⋂}}\left\{\left|\frac{b}{a}\sum_{i=1}^{a}y_{i}-\sum_{i=1}^{b}y_{i}\right|<\frac{a+b}{a}\lambda \right\}\right) & =P\left(\overset{a+c_{4}\Delta^{\alpha }}{\underset{b=a}{⋂}}\left\{\left|A_{a,b}\right|<\frac{a+b}{a}\lambda \right\}\right)\\ \; & =1-P\left(\overset{a+c_{4}\Delta^{\alpha }}{\underset{b=a}{⋃}}\left\{\left|A_{a,b}\right|\geq \frac{a+b}{a}\lambda \right\}\right)\\ \; & \geq 1-\sum_{b=a}^{a+c_{4}\Delta^{\alpha }}P\left(\left|A_{a,b}\right|\geq \frac{a+b}{a}\lambda \right)\\ \; & \geq 1-\sum_{b=a}^{a+c_{4}\Delta^{\alpha }}2\exp\left(-\frac{c_{5}{\left(\frac{a+b}{a}\lambda \right)}^{2}}{\frac{(b-a)b}{a}\sigma^{2}}\right)\\ \; & =1-\sum_{b=a}^{a+c_{4}\Delta^{\alpha }}2\exp\left(-\frac{c_{5}\lambda^{2}}{\sigma^{2}}\frac{{\left(a+b\right)}^{2}}{ab(b-a)}\right)\\ \; & \geq 1-2c_{4}\Delta^{\alpha }\exp\left(-c_{6}\frac{\lambda^{2}}{\Delta^{\alpha }}\right)\to 1, \end{array}$$

where the last inequality holds because $a,b\in \left[1,\tau_{1}\right],b-a\in \left[0,c_{4}\Delta^{\alpha }\right]$, and the final limit holds because $\lambda \geq c_{1}\sqrt{\Delta^{2-\alpha }\log\Delta }\geq c_{1}\sqrt{\Delta^{\alpha }\log\Delta }$.

Step 2: Prove there exists at least one $b=a+c_{7}\Delta^{\alpha }\in \left[1,\tau_{1}\right]$, $c_{7}<c_{4}$ such that

(A9) $$\lim_{n\to \infty }P\left(\left|\frac{b}{a}\sum_{i=1}^{a}y_{i}-\sum_{i=1}^{b}y_{i}\right|\leq \frac{b-a}{a}\lambda \right)=1.$$

From (A9), we can derive

$$\lim_{n\to \infty }P\left(\frac{\sum_{i=1}^{a}y_{i}-\lambda }{a}\leq \frac{\sum_{i=1}^{b}y_{i}-\lambda }{b}\right)=1,$$

and

$$\lim_{n\to \infty }P\left(\frac{\sum_{i=1}^{a}y_{i}+\lambda }{a}\geq \frac{\sum_{i=1}^{b}y_{i}+\lambda }{b}\right)=1.$$

Combining (A7) and (A9), we deduce the following: if $b_{i}^{-}=\left(\sum_{j=1}^{i-1}y_{j}-\lambda \right)/\left(i-1\right)$, then there exists at least one $k\in [i,i+c_{7}\Delta^{\alpha }]$ such that $b_{k}^{-}=\left(\sum_{j=1}^{k-1}y_{j}-\lambda \right)/\left(k-1\right)$; likewise, if $b_{i}^{+}=\left(\sum_{j=1}^{i-1}y_{j}+\lambda \right)/\left(i-1\right)$, then there also exists at least one $k\in [i,i+c_{7}\Delta^{\alpha }]$ such that $b_{k}^{+}=\left(\sum_{j=1}^{k-1}y_{j}+\lambda \right)/\left(k-1\right)$. Hence, there must exist $q_{1},q_{2}\in \left[\tau_{1}-c_{3}\Delta^{\alpha },\tau_{1}\right]$ such that $b_{q_{1}+1}^{-}=\left(\sum_{j=1}^{q_{1}}y_{j}-\lambda \right)/q_{1}$, $b_{q_{2}+1}^{+}=\left(\sum_{j=1}^{q_{2}}y_{j}+\lambda \right)/q_{2}$.

As n→∞,

$$\begin{array}{cc} P\left(\left|\frac{b}{a}\sum_{i=1}^{a}y_{i}-\sum_{i=1}^{b}y_{i}\right|\leq \frac{b-a}{a}\lambda \right) & =P\left(\left|\frac{a+c_{7}\Delta^{\alpha }}{a}\sum_{i=1}^{a}y_{i}-\sum_{i=1}^{a+c_{7}\Delta^{\alpha }}y_{i}\right|\leq \frac{c_{7}\Delta^{\alpha }}{a}\lambda \right)\\ \; & =P\left(\left|A_{a,a+c_{7}\Delta^{\alpha }}\right|\leq \frac{c_{7}\Delta^{\alpha }}{a}\lambda \right)\\ \; & \geq 1-2\exp\left(-\frac{c_{5}{\left(\frac{c_{7}\Delta^{\alpha }}{a}\lambda \right)}^{2}}{\frac{\left(a+c_{7}\Delta^{\alpha }\right)c_{7}\Delta^{\alpha }}{a}\sigma^{2}}\right)\\ \; & =1-2\exp\left(-\frac{c_{5}\lambda^{2}}{\sigma^{2}}\frac{c_{7}\Delta^{\alpha }}{a(a+c_{7}\Delta^{\alpha })}\right)\\ \; & \geq 1-2\exp\left(-c_{8}\frac{\lambda^{2}}{\Delta^{2-\alpha }}\right)\to 1, \end{array}$$

where the last inequality holds because $a\in [1,\tau_{1}]$, and the final limit holds because $\lambda \geq c_{1}\sqrt{\Delta^{2-\alpha }\log\Delta }$.

Step 3: Prove that, when $\theta_{\tau_{1}+1}-\theta_{\tau_{1}}<0$, there exists at least one $b=\tau_{1}+c_{3}\Delta^{\beta }$ such that

(A10) $$\lim_{n\to \infty }P\left(\frac{\sum_{i=1}^{q_{1}}y_{i}-\lambda }{q_{1}}\geq \frac{\sum_{i=1}^{b}y_{i}+\lambda }{b}\right)=1,$$

where $q_{1}\in \left[\tau_{1}-c_{3}\Delta^{\alpha },\tau_{1}\right]$ is the last position that satisfies $b_{q_{1}+1}^{-}=\left(\sum_{j=1}^{q_{1}}y_{j}-\lambda \right)/q_{1}$. (A10) ensures that there must exist $q_{3}\in \left[\tau_{1}+1,\tau_{1}+c_{3}\Delta^{\beta }\right]$ such that all the information about $y_{1},y_{2},\ldots ,y_{q_{3}-2}$ is no longer present in $g_{q_{3}}^{\prime }\left(x\right)$, similar to what occurs in (i).

Similar to Step 1, we have $E\left(A_{q_{1},b}\right)=\left(b-\tau_{1}\right)\delta$ for $b>\tau_{1}$. As n→∞,

$$\begin{array}{cc} \; & P\left(\frac{\sum_{i=1}^{q_{1}}y_{i}-\lambda }{q_{1}}\geq \frac{\sum_{i=1}^{b}y_{i}+\lambda }{b}\right)\\ = & P\left(\frac{\sum_{i=1}^{q_{1}}y_{i}-\lambda }{q_{1}}\geq \frac{\sum_{i=1}^{\tau_{1}+c_{3}\Delta^{\beta }}y_{i}+\lambda }{\tau_{1}+c_{3}\Delta^{\beta }}\right)\\ = & P\left(A_{q_{1},\tau_{1}+c_{3}\Delta^{\beta }}-c_{3}\Delta^{\beta }\delta \geq \frac{\tau_{1}+c_{3}\Delta^{\beta }+q_{1}}{q_{1}}\lambda -c_{3}\Delta^{\beta }\delta \right)\\ \geq & 1-2\exp\left(-\frac{c_{5}{\left(\frac{\tau_{1}+c_{3}\Delta^{\beta }+q_{1}}{q_{1}}\lambda -c_{3}\Delta^{\beta }\delta \right)}^{2}}{\frac{\left(\tau_{1}+c_{3}\Delta^{\beta }\right)\left(\tau_{1}+c_{3}\Delta^{\beta }-q_{1}\right)}{q_{1}}\sigma^{2}}\right)\\ \geq & 1-2\exp\left(-c_{9}\frac{\Delta^{2\beta }\delta^{2}}{c_{10}\Delta^{\alpha }+c_{11}\Delta^{\beta }}\right)\to 1, \end{array}$$

where the second inequality holds because $\lambda \leq c_{2}\Delta^{\beta }\delta$, and the final limit holds because $\Delta^{2\beta -\max(\alpha ,\beta )}\delta^{2}\to \infty$ when n→∞. Similarly, we can prove that, when $\theta_{\tau_{1}+1}-\theta_{\tau_{1}}>0$, there exists at least one $b=\tau_{1}+c_{3}\Delta^{\beta }$ such that

(A11) $$\lim_{n\to \infty }P\left(\frac{\sum_{i=1}^{q_{2}}y_{i}+\lambda }{q_{2}}\leq \frac{\sum_{i=1}^{b}y_{i}-\lambda }{b}\right)=1,$$

where $q_{2}\in \left[\tau_{1}-c_{3}\Delta^{\alpha },\tau_{1}\right]$ is the last position that satisfies $b_{q_{2}+1}^{+}=\left(\sum_{j=1}^{q_{2}}y_{j}+\lambda \right)/q_{2}$. (A11) ensures that there must exist $q_{3}\in \left[\tau_{1}+1,\tau_{1}+c_{3}\Delta^{\beta }\right]$ such that all the information about $y_{1},y_{2},\ldots ,y_{q_{3}-2}$ is no longer present in $g_{q_{3}}^{\prime }\left(x\right)$, similar to what occurs in (v).

In summary, Step 1 and Step 2 guarantee that there exist $q_{1},q_{2}\in \left[\tau_{1}-c_{3}\Delta^{\alpha },\tau_{1}\right]$ such that $b_{q_{1}+1}^{-}=\left(\sum_{j=1}^{q_{1}}y_{j}-\lambda \right)/q_{1}$, $b_{q_{2}+1}^{+}=\left(\sum_{j=1}^{q_{2}}y_{j}+\lambda \right)/q_{2}$. Moreover, we have $b_{i}^{-}<b_{q_{1}+1}^{-}$ for all $i\leq q_{1}$ and $b_{j}^{+}>b_{q_{2}+1}^{+}$ for all $j\leq q_{2}$. Combining (A4), we obtain

$$\lim_{n\to \infty }P\left({\hat{\theta }}_{1}=\ldots ={\hat{\theta }}_{\tau_{1}-c_{3}\Delta^{\alpha }}\in \left[b_{q_{1}+1}^{-},b_{q_{2}+1}^{+}\right]\right)=1.$$

Notably, since $\lambda \leq c_{2}\Delta^{\beta }\delta$ and $\delta =o(\Delta^{1-\beta })$, it follows that

$$\frac{\lambda }{\min(q_{1},q_{2})}=O\left(\frac{\lambda }{\Delta }\right)=O\left(\Delta^{\beta -1}\delta \right)=o\left(1\right).$$

Step 3 guarantees that there exists $q_{3}\in \left[\tau_{1}+1,\tau_{1}+c_{3}\Delta^{\beta }\right]$ such that all the information about $y_{1},y_{2},\ldots ,y_{q_{3}-2}$ is no longer present in $g_{q_{3}}^{\prime }\left(x\right)$ while simultaneously guaranteeing that

$$\lim_{n\to \infty }P\left({\hat{\theta }}_{\tau_{1}-c_{3}\Delta^{\alpha }}=\ldots ={\hat{\theta }}_{\tau_{1}+c_{3}\Delta^{\beta }}\right)=0.$$

Furthermore, the interval $\left[\tau_{1}+c_{3}\Delta^{\beta },n\right]$ has the same properties as the interval $\left[1,\tau_{1}\right]$. Consequently, by following a proof that is analogous to Step 1 and Step 2, we obtain

$$\lim_{n\to \infty }P\left({\hat{\theta }}_{\tau_{1}+c_{3}\Delta^{\beta }}=\ldots ={\hat{\theta }}_{n}\right)=1.$$

### Appendix B.2. The Proof of Theorem 3

The proof of Theorem 3 follows the same strategy as that of Theorem 2. Since intervals $\left[\tau_{i}+c_{3}\Delta^{\beta },\tau_{i+1}\right]$ for i=1,…,m have the same properties as the interval $\left[1,\tau_{1}\right]$, the reasoning from Step 1 and Step 2 of Theorem 2 applies directly to them. Moreover, the reasoning in Step 3 of Theorem 2 applies equally to all the change-points $\tau_{1},\ldots \tau_{m}$. Let γ=max(α,β), and then (A4) holds.
